# Life span of different extracorporeal membrane systems for severe respiratory failure in the clinical practice

**DOI:** 10.1371/journal.pone.0198392

**Published:** 2018-06-01

**Authors:** Alois Philipp, Filip De Somer, Maik Foltan, Andre Bredthauer, Lars Krenkel, Florian Zeman, Karla Lehle

**Affiliations:** 1 Department of Cardiothoracic Surgery, University Hospital Regensburg, Regensburg, Germany; 2 Heart Centre 5K12, University Hospital Ghent, Ghent, Belgium; 3 Department of Anesthesiology, University Hospital Regensburg, Regensburg, Germany; 4 Regensburg Center of Biomedical Engineering, Ostbayerische Technische Hochschule, Regensburg, Germany; 5 Center for Clinical Studies, University Hospital Regensburg, Regensburg, Germany; University of Bern, University Hospital Bern, SWITZERLAND

## Abstract

Over the past decade, veno-venous extracorporeal membrane oxygenation (vvECMO) has been increasingly utilized in respiratory failure in patients. This study presents our institution´s experience focusing on the life span of ECMO systems reflecting the performance of a particular system. A retrospective review of our ECMO database identified 461 adult patients undergoing vvECMO (2010–2017). Patients that required more than one system and survived the first exchange >24 hours (n = 139) were included. Life span until the first exchange and exchange criteria were analyzed for all systems (PLS, Cardiohelp HLS-set, both Maquet Cardiopulmonary, Rastatt, Germany; Deltastream/Hilite7000LT, iLA-activve, Xenios/NovaLung, Heilbronn, Germany; ECC.O5, LivaNova, Mirandola, Italy). At our ECMO center, the frequency of a system exchange was 30%. The median (IQR) life span was 9 (6–12) days. There was no difference regarding the different systems (p = 0.145 and p = 0.108, respectively). However, the Deltastream systems were exchanged more frequently due to elective technical complications (e. g. worsened gas transfer, development of coagulation disorder, increased bleedings complications) compared to the other exchanged systems (p = 0.013). In summary, the used ECMO systems are safe and effective for acute respiratory failure. There is no evidence for the usage of a specific system. Only the increased predictability of an imminent exchange preferred the usage of a Deltastream system. However, the decision to use a particular system should not depend solely on the possible criteria for an exchange.

## Introduction

Veno-venous extracorporeal membrane oxygenation (vvECMO) has been increasingly used for potentially reversible severe refractory acute respiratory failure associated with severe influenza A (H1N1) pneumonia [1–3]. Still, it remains an expensive, resource-intensive procedure with significant complications, but with a promising survival rate justifying its increasing use [4–7].

Several devices are available for clinical use. At our ECMO center five different ECMO-systems are available [8–10]. The choice of a specific device is based upon clinical considerations and preferences and by the availability of equipment in the unit. All systems were safe and effective for short-term support as a bridge to recovery and/or as bridge to transplantation. However, up to now long-term application as an artificial lung failed due to the development of coagulation disorders and thrombus formation [11,12].

A number of equivalent ECMO devices are available on the market. The appearance of technical-induced complications and the requirement for a system exchange limited the life span of an ECMO system [12]. To our knowledge there was no information about the performance of a particular system in critically ill patients.

## Materials and methods

### Patients and ECMO systems

Between January 2010 and November 2017, 461 adult patients with severe respiratory failure were treated with conventional ECMO systems [PLS-system (PLS, n = 117), Cardiohelp HLS-set (CH, n = 107), both Maquet Cardiopulmonary, Rastatt, Germany; Deltastream-system/Hilite7000LT (HL, n = 135), iLA-activve (n = 15), Xenios/NovaLung, Heilbronn, Germany; ECC.O5-system (ECC.05, n = 87), LivaNova, Mirandola, Italy] (S1 Table) [3–6]. Only patients that required more than one system and survived the first exchange >24 hours (n = 139) were included. The life span (LS) of a system was defined as the period from initiation to exchange due to technical complications [12]. The LS was used to estimate the performance of a particular system. Only the time period from initiation to the first exchange was analyzed. The total ECMO support described the time from the initiation of the first system to the end of ECMO therapy. The assignment to a specific system was based on clinical availability. The retrospective analysis was approved by the Ethics Committee of the University of Regensburg (vote-no. 167-101-0322). Informed consent requirement was waived due to its retrospective nature. All devices had been approved for clinical use, no personalized data were used.

The indications for vvECMO in acute respiratory failure have been reported in detail earlier [6,9]. The technique of vvECMO has been described in detail elsewhere [13–15]. All available ECMO systems were equipped with a polymethylpentene (PMP) membrane oxygenator (S1 Table). After a bolus of unfractionated heparin (1000–5000 IE heparin according to impairment of coagulation), systemic anticoagulation was continued with unfractionated heparin aiming at an activated partial thromboplastin time (aPTT) of 50–60 s for all devices. Heparin was temporarily stopped if significant bleeding occurred. A hemoglobin (HB) concentration of > 8.0 g/dL was targeted, with exception of patients with borderline oxygenation despite ECMO (HB concentration > 12 g/dL). Platelets were transfused below a count of 20,000/μL, or in case of bleeding. Fresh frozen plasma (FFP) was substituted if bleeding due to DIC (disseminated intravascular coagulation) occurred or in the course of plasmapheresis [13].

### Exchange criteria

Exchange of an ECMO system occurred in 139 patients. The reason for a system exchange was documented in our database (Regensburg ECMO Registry). Technical complications demanding an acute system-exchange included mechanical/technical failure (MF) and acute clot formation within the oxygenator (acute oxygenator thrombosis, AOT) or pump head (pump head thrombosis, PHT) [12]. MF was defined as a malfunction of the pump head, the control module or the sensor system as well as air, blood or water leakage of the oxygenator. AOT caused an increase in the transmembrane pressure drop (dpMO) and a decrease in blood flow at the same pump speed [16]. Sudden sound changes in the pump head, technically induced hemolysis [11,17] and a decrease in platelet count indicates PHT. Furthermore, technical complications demanding an elective system exchange were identified as an announcing impairment of the gas transfer (GT) capability of the oxygenator, the development of coagulation disorder (CD) and diffuse bleeding events [12]. Restricted GT goes along with an increase in gas flow rates with worsened/unchanged gas transfer. CD was indicated by an otherwise unexplainable increase in D-dimer levels from < 10 mg/dL to 25–35 mg/dL [18] and a decrease in fibrinogen levels (< 200 mg/dL). Elective reasons for a system exchange based always on a combination of CD, GT or increased risk for diffuse bleeding.

### Data collection and statistical analysis

All data from patients treated with vvECMO were collected prospectively in the Regensburg ECMO Registry. System exchange frequency was documented. The life span of the different ECMO systems was analyzed for patients that were treated with more than one system during ECMO support (n = 139). In addition, the reasons for a system exchange (see above) were documented.

Statistical analysis was performed with the statistic package SigmaStat3.5 (Systat Software, Erkrath, Germany). Continuous data are presented as median and interquartile range (IQR) and were compared between groups by using the non-parametric Kruskal-Wallis test. Categorical data are presented as absolute and relative frequencies and were compared using a Chi-squared test of independence. Significance was assumed for a p-value <0.05. Due to the explorative character of the study, no adjustment of the level of significance for multiple statistical analyses was performed.

## Results

### Frequency of a system exchange

The overall frequency of a system exchange was 30% (139/461). No significant difference could be found between the used ECMO systems (p = 0.145; Table 1), while HL showed the lowest frequency with 24% and iLA-activve the highest with 40%. Predictably, the total ECMO support of patients that required more than one system was significantly prolonged [median (IQR), 19 (13–27) days] compared to patients with only one system [median (IQR), 7 (4–10) days; p<0.001]. While the median time from initiation to the 1^st^ exchange (= LS) was 9 (6–12) days, the ECMO time from patients with only one system was significantly shortened [median (IQR), 7 (4–10) days; p<0.001].

**Table 1:** Total EMCO support time and life span of the first ECMO system.

	All	PLS	CH	HL	ECC.O5	iLA-activve	p-value^§^
**all ECMO runs (n)**	461	117	107	135	87	15	
**exchanged ECMO runs, n (%)**	139 (30%)	35 (30%)	41 (38%)	32 (24%)	25 (29%)	6 (40%)	0.145^**Chi**^
**Number of oxygenators (min-max)**	2–7	2–4	2–7	2–7	2–5	2–4	0.531 ^**KW**^
**Total ECMO support (days), median (IQR)**	19 (13–27)	17 (13–25)	16 (10–24)	24 (15–34)	21 (13–29)	19 (8–32)	0.063 ^**KW**^
**LS (days), median (IQR)**	9 (6–12)	8 (7–12)	7 (5–12)	10 (7–15)	9 (7–11)	7 (3–15)	0.108 ^**KW**^

LS, life span, time from initiation of a system until the first exchange.

^**KW**^, Kruskal-Wallis test;

^**Chi**^, Chi-squared test.

The requirement of a system exchange resulting from device-induced technical complications reflected the persistence of the used ECMO systems. Therefore, the LS of the particular ECMO systems were analyzed. The median LS of the systems ranged between 7 (CH and iLA-activve) and 10 days (HL) (Table 1) whereas no statistically significant difference could be found (p = 0.108). S2 Table listed the LS, total ECMO support, number of used ECMO systems per patient, and the reasons for the first exchange of the included ECMO systems.

### Reasons for a system exchange

**Fig 1:**
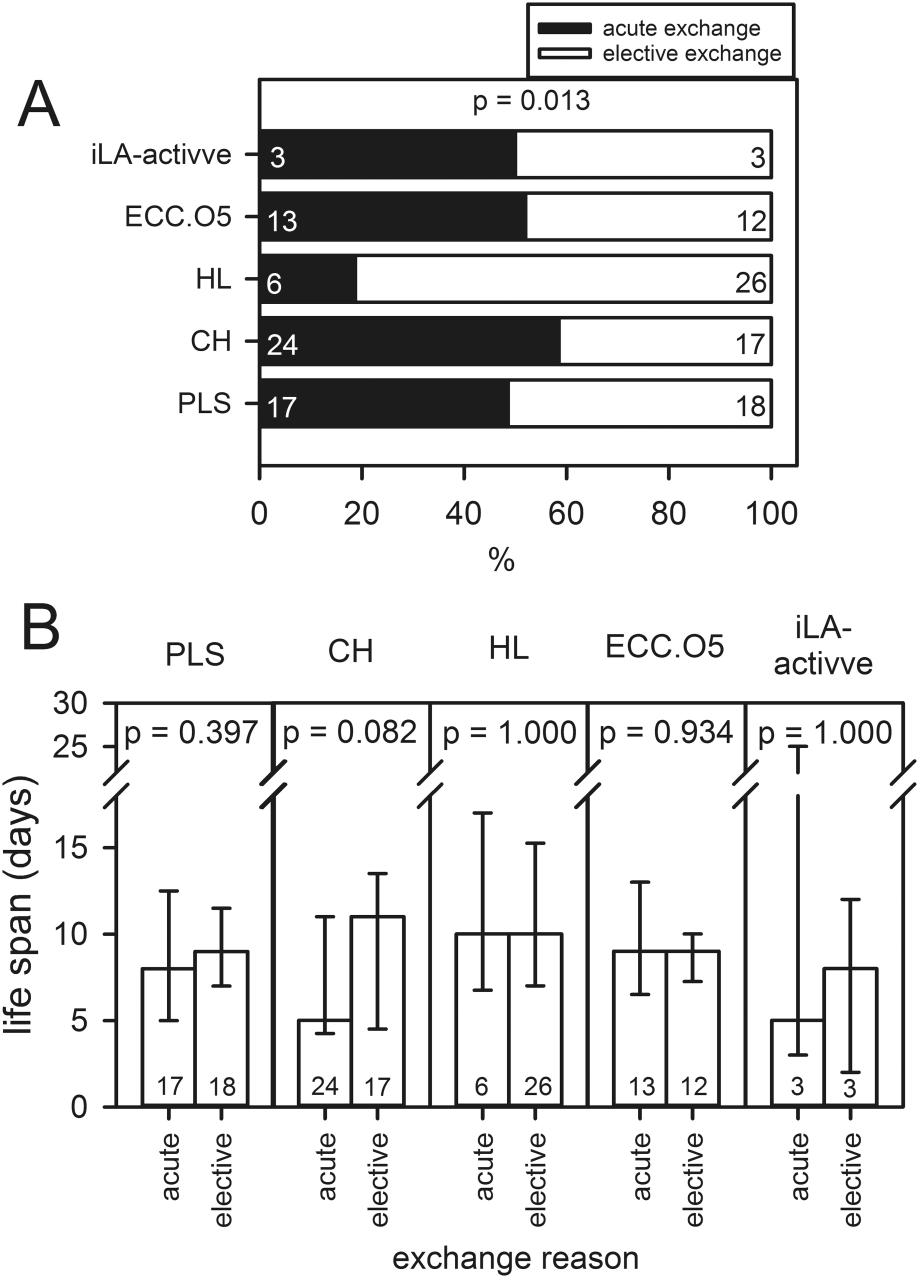
Reasons for a system exchange. Exchange criteria from 139 ECMO patients that required a system exchange including data from five different ECMO systems (PLS, CH, HL, ECC.O5, iLA-activve, see S1 and S2 Tables). (A) Proportion (in %) of acute (MF, mechanical failure; AOT, acute oxygenator thrombosis; PHT, pump head thrombosis) (filled bars) and elective (GT, worsened gas transfer; CD, coagulation disorder; bleeding diathesis) exchange reasons (white bars). (B) Median (IQR) of the life span of acute and elective exchanges of the different ECMO systems. P-values compared the life span of acute and elective exchanges. The numbers within the bars represent the number of exchanges.

Technical complications demanding an acute system exchange occurred due to MF, AOT and PHT in 22 (5% of all ECMO runs), 16 (3% of all ECMO runs) and 25 (5% of all ECMO runs) cases, respectively. In contrast, 76 systems (16% of all ECMO runs) were exchanged due to restricted GT and/or CD. Regarding the different ECMO systems, a significant difference between the proportions of acute and elective exchanges could be found (p = 0.013). Pairwise comparisons revealed significant differences between the HL system (only 19% of exchanged HL systems were replaced due to development of acute technical complications) and ECC.O5 (52%, p = 0.008), CH (59%, p<0.001), PLS (49%, p = 0.010) (Fig 1a). Notwithstanding this difference, the life spans of PLS, HL, ECC.O5 and iLA-activve that were exchanged due to acute or elective events were comparable, while the life span of CH seemed to be prolonged for elective events [median (IQR): 11 (5–14) vs. 5 (4–11) days; p = 0.082] (Fig 1b).

Acute events are associated with a high risk to the patient and required immediate intervention by healthcare professionals. Table 2 listed all acute events for the different systems. A striking feature was the high proportion of MF of the acute PLS (10/17, 59%) and HL (3/6, 50%) system exchanges. One third of it affected the oxygenators in form of a blood leakage from the gas exhaust ports. The remaining MF descended from failures of the blood pumps caused by electrical failure of the pump drive (HL, n = 1; PLS, n = 5) or the control module (HL, n = 1; PLS, n = 1) and material defects of the pump head (PLS, n = 1). The MF of the affected CH systems consisted of a material defect of the pump head (n = 1), a technical fault of the integrated sensors (n = 2), and air leakage into the circuit due to occluded cannulas (n = 1). MF of the remaining systems was caused by material defects of the pump heads (iLA-activve, n = 1; ECC.O5, n = 2) and a mechanical defect of a cannula (ECC.O5, n = 1). The proportion of AOT was not different regarding the different systems, except only one AOT for a PLS oxygenator. Only one Deltastream blood pump presented a PHT, while the frequency of a PHT was higher for the blood pumps from the other systems. There was no statistical difference comparing the proportion of MF, AOT and PHT between the different systems (Table 2, p = 0.275).

**Table 2:** Details of acute exchange reasons for the different systems.

	All	PLS	CH	HL	ECC.O5	iLA-activve	p-value
**Acute exchanges (n)**	63	17	24	6	13	3	
**AOT, n (%)**	**16 (25%)**	**1 (6%)**	**8 (33%)**	**2 (33%)**	**4 (31%)**	**1 (33%)**	**0.275**^**Chi**^
**PHT, n (%)**	**25 (40%)**	**6 (35%)**	**11 (46%)**	**1 (17%)**	**6 (46%)**	**1 (33%)**	
**MF, n (%)**	**22 (35%)**	**10 (59%)**	**5 (21%)**	**3 (50%)**	**3 (23%)**	**1 (33%)**	
*Cannula*, *n (%)*	*2 (9%)*	*0 (0%)*	*1 (20%)*	*0 (0%)*	*1 (33%)*	*0 (0%)*	
*Pump*, *n (%)*	*13 (59%)*	*7 (70%)*	*1 (20%)*	*2 (67%)*	*2 (67%)*	*1 (100%)*	
*Oxygenator*, *n (%)*	*5 (23%)*	*3 (30%)*	*1 (20%)*	*1 (33%)*	*0 (0%)*	*0 (0%)*	
*Others*, *n (%)*	*2 (9%)*	*0 (0%)*	*2 (40%)*	*0 (0%)*	*0 (0%)*	*0 (0%)*	

AOT, acute oxygenator thrombosis; PHT, pump head thrombosis; MF, mechanical failures were subdivided regarding the affected circuit compounds. Statistics compared the differences between AOT, PHT and MF of the different systems (^**Chi**^, Chi-squared test).

## Discussion

Despite improvement in vvECMO technology and management, the most common complication remains clot formation within the ECMO circuit that required a system exchange and restricted long-term usage [19]. In this study the frequency of a system exchange was 30%. The different systems were exchanged after a median (IQR) life span of 9 (6/12) days. However, in our patient population there were less acute events during usage of a HL system resulting in a system exchange. Instead it was mostly exchanged due to worsened GT and/or CD.

It is well known that blood cell contact with membrane oxygenator surfaces activate the coagulation system, platelets and the inflammatory system resulting in device-induced coagulation disorder, clot formation and, in the worst case, requirement of a system exchange [12]. These events as well as technical failures of circuit components limit the life span of currently available ECMO systems. Approved times of usage ranged between 1 and 30 days according to manufacturers´ instruction. However, in critically ill patients the median exposure times of all analyzed ECMO systems were 9 (6/12) days. Neither differences in the circuit design, the gas exchange areas, the surface coatings nor the types of blood pumps (S1 Table) predestined the usage of a particular system. Obviously, the consistent life span of the different systems derived from the strict management at our ECMO center. That includes the daily inspection of the system components, the control of the gas exchange capacity and the pressure drop over the oxygenator as well as assessment of coagulation and hemolysis parameters [9–12]. This procedure allowed early identification of technical complications [12]. The ECMO management was independent of the ECMO type and patient.

However, as a consequence of this practice every 3^rd^ system was exchanged. Notably, the special focus on a worsened gas exchange capability of the oxygenator, the appearance of hemolysis as well as control of increased bleeding events increased the amount of system exchanges. However, the opportunity to predict a system thrombosis is a valuable tool to prevent high risk incidence of acute technical complications such as AOT or PHT. Acute events are always associated with a high risk to the patient and required immediate day and night intervention by healthcare professionals [12].

The retrospective analysis of the reasons for a system exchange revealed that the exchanged HL systems were mainly replaced due to elective reasons. Only 19% of the exchanged HL systems showed acute events. It was surprising that only one HL system showed a PHT compared to the centrifugal pumps from the exchanged PLS (6 of 17) or CH (11 of 24) systems. Due to low sample size of acute events statistical analysis was not executed. In the literature, only single-center pediatric cohort studies with low sample sizes reported on low incidence of technical failures using the DP3 diagonal pumps as part of the HL systems [20,21]. Future multi-centric studies are required to investigate thrombogenesis in the DP3 blood pumps on ECMO. More than 80% of the exchanged HL systems were exchanged due to the development of circuit-induced coagulation disorders with subsequent worsening of the gas transfer/elimination efficacy of the HL oxygenator. A clear demarcation of the two exchange reasons is critical. However, the elective exchange reasons allowed the prediction of an imminent system exchange and replacement of the system during regular working hours with sufficient resources available and not on an emergency basis. Overall, the development of a coagulation disorder may cause thrombogenesis resulting in cellular accumulations on the surface of the gas exchange membranes within the oxygenators and a subsequent increase in the diffusion distance that will reduce gas transfer capacity [16]. A systematic analysis of the extent of surface coverage within different oxygenators failed so far. This is part of an ongoing study. Preliminary data described uniformly distributed clots within PLS oxygenators [22–24]. Data on other oxygenator types failed. Ultimately, it is not decisive whether the development of thrombosis can be observed by means of biomarkers or imaging procedures, or whether a rapid occlusion of the blood-bearing spaces occurs.

Differences in oxygenator design might be another partial explanation for the reported differences. Hilite 7000LT and ECC.O5 oxygenators have a small frontal area of 14 cm^2^ and 16.5 cm^2^, respectively. Both Quadrox PLS and CH oxygenators have a much larger frontal area of 81 cm^2^ and iLA-activve has the largest with 90 cm^2^. As a consequence the average velocity and average pressure drop over the fiber bundle is higher in designs with a lower frontal area [25]. The advantage of such an approach is a better distribution of the blood flow over the total membrane surface and a lower risk for local stasis and or recirculation. A disadvantage is the fact that a small amount of formed clot will result in fast increase in pressure drop over the design compared to designs with a larger frontal area [9]. The higher incidence of PHT with the potential for thrombus translocation towards the oxygenator in combination with the small frontal area might explain the higher incidence of acute exchange in the ECC.O5. More intriguing is the difference between CH and PLS. Both oxygenators share the same design with exception of the design of the venous inlet. In the PLS the venous inlet connector is placed in the lower corner with small distance to a baffle plate. In the CH there are four inlet connectors at the center of the oxygenator. Future studies will analyze the flow regime within oxygenators using Computational Fluid Dynamics (CFD). We suppose that the inlet position, the geometry and the flow rate may influence the resulting flow regime dramatically leading to significant changes in shear rate and potentially generates adverse recirculation or low- or high-flow areas.

All these aspects are well known to induce coagulation or flow induced cell activation. The iLA-activve has the lowest velocities and shear rates in the membrane compartment compared to all other designs. On the one hand side, this is due to the large frontal area and on the other hand by the lower blood flows (average 1.8 L/min). The combination of low velocity (0.33 cm/s) with low shear (6.2 dynes/cm^2^) augments the risk for platelet agglomeration and adhesion to the foreign material [26].

In summary, there is no preference for a specific ECMO system. The decision for usage of a particular system especially for new developing ECMO centers or in emerging countries when the family of a patient has to decide about the continuation of the expensive ECMO therapy should include the individual requirements for the patients.

Finally, the prevention of thrombogenesis on the polymeric surfaces and knowledge about the real flow dynamics within systems are the ultimate goals of future studies to generate devices for complete or paracorporeal implantation as an artificial lung (Current Priority Program of the DFG, Deutsche Forschungsgemeinschaft, entitled “Towards an implantable Lung”).

Limitations of this study include its retrospective character, the low sample size for the ECC.O5 and the iLA-activve systems, and the data collection from a single center. A further limitation might also be the non-classified patient group covered within this overview study. From clinical aspects it would be interesting to differentiate between different primary diseases which result in heterogeneous immune activation and coagulation responses. However, this is covered in recent work of our group.

## Conclusions

All currently available ECMO systems are safe and effective for acute respiratory failure. The life spans of these extracorporeal systems are independent of the circuit design. Only the increased predictability of an imminent acute exchange preferred the usage of a Deltastream system. However, the limited life span of the systems used in critically ill patient prevented long-term usage as an artificial lung. The decision to use a particular system should not depend solely on the possible criteria for an exchange.

## Supporting information

S1 Table Available ECMO devices for clinical usage. (DOCX)

S2 Table Life span and exchange reasons of the first ECMO system. (XLSX)
