# Supplementary material for: Life span of different extracorporeal membrane systems for severe respiratory failure in the clinical practice
Source: PLoS One. 2018 Jun 1;13(6):e0198392. doi: 10.1371/journal.pone.0198392 (PMC5983427; doi:10.1371/journal.pone.0198392)
Supplement: S1 Table — (DOCX) [file pone.0198392.s001.docx]

**S1 Table.** Available ECMO devices for clinical usage.

| ECMO system | PLS-system | Cardiohelp HLS-set | Deltastream-system/Hilite7000LT | ECC.O5 | iLA-activve |
| --- | --- | --- | --- | --- | --- |
| Abbreviation | PLS | CH | HL | ECC.O5 | ILA |
| Manufacturer | Maquet | Maquet | Xenios | Sorin | Xenios/NovaLung |
| Max. blood flow rate (L/min) | 0.5-7.0 | 0.5-7.0 | 1.0-7.0 | 1.0-5.0 | 0.5-4.5 |
| Main applications | CO_2_ removal / oxygenation | CO_2_ removal / oxygenation | CO_2_ removal / oxygenation | CO_2_ removal / partial oxygenation | CO_2_ removal / partial oxygenation |
| GEM: Material/Surface (m^2^) | PMP/1.8 | PMP/1.8 | PMP/1.9 | PMP/1.2 | PMP/1.3 |
| HE: Material/Surface (m^2^) | PUR/0.60 | PUR/0.60 | PET/0.45 | Steel/0.14 | No |
| Surface coating | Bioline® | Bioline® | Rheoparin® | Phisio® | x.ellence® |
| Blood pump | Rotaflow (centrifugal pump) | Rotaflex ^a^ (centrifugal pump) | Deltastream, DP3 (diagonal pump) | Revolution5 ^a^ (centrifugal pump) | Deltastream, DP3 (diagonal pump) |
| System approval (days) | 14 | 30 | 1 | 5 | 29 |
| Transport approval ^b^ | No | Yes | No | Yes | No |

^a^, integrated blood pumps; ^b^, ambulance or air transport; GEM, gas exchange membrane; HE, heat exchanger; PMP, polymethylpentene; PUR, polyurethane. Maquet Cardiopulmonary, Rastatt, Germany; Xenios/NovaLung, Heilbronn, Germany; Sorin Group, Modena, Italy.
